# PD-1 Independent Role of PD-L1 in Triple-Negative Breast Cancer Progression

**DOI:** 10.3390/ijms24076420

**Published:** 2023-03-29

**Authors:** Duaa Alkaabi, Kholoud Arafat, Shahrazad Sulaiman, Aya Mudhafar Al-Azawi, Samir Attoub

**Affiliations:** 1 Department of Pharmacology & Therapeutics, College of Medicine & Health Sciences, United Arab Emirates University, Al-Ain 15551, United Arab Emirates; 2 Institut National de la Santé et de la Recherche Médicale (INSERM), 75013 Paris, France

**Keywords:** PD-L1, TNBC, proliferation, migration, invasion, CAM, Akt, ERK

## Abstract

Triple-negative breast cancer (TNBC) is a type of breast malignancy characterized by a high proliferative rate and metastatic potential leading to treatment failure, relapse, and poor prognosis. Therefore, efforts are continuously being devoted to understanding its biology and identifying new potential targets. Programmed death-ligand 1 (PD-L1) is an immunosuppressive protein that inactivates T cells by binding to the inhibitory receptor programmed death-1 (PD-1). PD-L1 overexpression in cancer cells contributes to immune evasion and, subsequently, poor survival and prognosis in several cancers, including breast cancer. Apart from its inhibitory impact on T cells, this ligand is believed to have an intrinsic role in cancer cells. This study was performed to clarify the PD-1 independent role of PD-L1 in TNBC MDA-MB-231 cells by knocking out the PD-L1 using three designs of CRISPR-Cas9 lentiviral particles. Our study revealed that PD-L1 knockout significantly inhibited MDA-MB-231 cell proliferation and colony formation in vitro and tumor growth in the chick embryo chorioallantoic membrane (CAM) model in vivo. PD-L1 knockout also decreased the migration and invasion of MDA-MB-231 cells in vitro. We have shown that PD-L1 knockout MDA-MB-231 cells have low levels of p-Akt and p-ERK in addition to some of their downstream proteins, c-Fos, c-Myc, p21, survivin, and COX-2. Furthermore, PD-L1 knockout significantly decreased the expression of Snail and RhoA. This study shows the intrinsic role of PD-L1 in TNBC independently of its binding to PD-1 receptors on T cells. It may pave the way for developing novel therapeutic strategies using PD-L1 inhibitors alone and in combination to treat TNBC more effectively.

## 1. Introduction

The global cancer incidence is estimated by the World Health Organization at 19.3 million new cases and 10 million deaths in 2020, making cancer the second highest cause of death worldwide [1]. Breast cancer (BC) is the most common cancer with the highest mortality rate in women worldwide, accounting for 2.3 million cases and 0.68 million deaths in 2020 [1]. TNBC is the most aggressive breast cancer, accounting for 15–20% of all breast cancer patients and a 40% mortality rate within 5 years after diagnosis [2]. Its prognosis is very poor, mainly because of its high metastasis incidence and low efficacy of the current management protocols [2,3]. A worse overall survival (OS) in BC patients was associated with positive expression of PD-L1 [4].

PD-L1, also known as CD274 or B7-H1, is a 40 kDa transmembrane protein and a member of the B7/CD28 family of proteins that control T-cell activation. PD-L1 expression is regulated by transcriptional factors (STATs, NF-κB, AP-1, and HIF-1α) that bind to the gene promoter. The extracellular signals (hypoxia, cytokines, and EGF signals) will be transduced via different pathways (mainly through Ras/MAPK and/or PI3K/Akt) to regulate PD-L1 expression at the transcriptional level [5,6,7]. PD-L1 is typically expressed on antigen-presenting cells, while expression can be detected in other non-immune cells such as mesenchymal stem cells, epithelial cells, vascular endothelium, bone marrow-derived mast cells, and tumor cells, including melanoma, renal carcinoma, lung cancer, and breast cancer [5,8,9]. It is the ligand for the PD-1 checkpoint receptor on the surface of immune cells and mainly T lymphocytes [10]. The binding of PD-L1 to its receptor PD-1 forms a biochemical “shield”, protecting tumor cells from being destroyed by the lymphocytes, which promotes cancer progression [11]. PD-L1 and PD-1 immune checkpoint antibodies-based immunotherapy are gaining massive momentum in oncology, showing great promise in cancer management [12,13].

Many tumors can upregulate the expression of PD-L1, inhibiting anti-tumor T-cell responses and avoiding immune surveillance and elimination. Furthermore, it has been found that the expression of PD-L1 is associated with shorter survival and poorer prognosis in many cancers, including BC [4,14]. TNBC is a highly aggressive metastatic cancer with a very high mortality rate [2], and 50% of these tumors showed a high level of PD-L1 expression [15]. The analysis of PD-L1 expression in lymph node-positive TNBC patients suggests that PD-L1 is expressed significantly higher in tumor cells that metastasize to the lymph nodes [16]. It has also been reported that the PD-L1 mRNA and protein levels in the ER-positive breast cancer cell lines MCF7 and T47D cells were markedly lower compared with those in the ER-negative breast cancer cell lines BT549 and MDA-MB-231 cells [17]. In light of the recent literature, it is suggested that the role of PD-L1 is not only restricted to its interaction with PD-1 receptors on the surface of the lymphocytes. In fact, independent of its interaction with PD-1, PD-L1 is suggested to control tumor progression [13].

In order to clarify the PD-L1 role in TNBC tumor growth and metastasis, we established a PD-L1 knockout in highly tumorigenic and metastatic TNBC cells MDA-MB-231 using CRISPR-Cas9 lentiviral particles technology. The impact of PD-L1 knockout was investigated on cell proliferation, colony growth, migration and invasion in vitro, and on tumor growth in vivo using chick embryo CAM assay. In addition, the effect of PD-L1 knockout on the expression and activity of major cancer-targeted genes was also determined.

## 2. Results and Discussion

### 2.1. PD-L1 Knockout in TNBC MDA-MB-231 Cell Line

**Figure 1:**
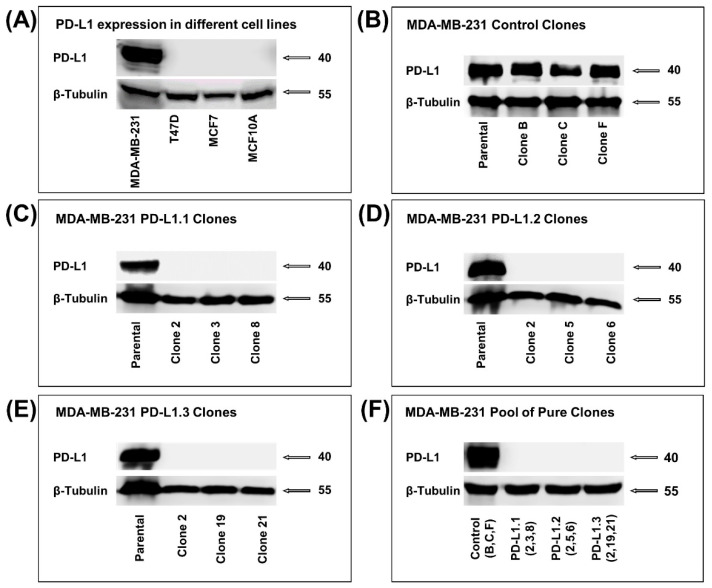
PD-L1 knockout in MDA-MB-231 cell line using CRISPR/Cas9. (**A**) Western blot showing the level of PD-L1 in different breast cell lines. (**B**–**E**) Western blot showing the level of PD-L1 in the pure clones of the control, PD-L1.1, PD-L1.2, and PD-L1.3. (**F**) Western blot showing PD-L1 level in the pool of the three selected pure clones from the control and each design of sgRNA targeting PD-L1.

In the present study, we first investigated PD-L1 expression levels in four different breast cell lines. The TNBC cells, MDA-MB-231, showed a very high PD-L1 expression with almost no expression in two human hormone-dependent breast cancer cell lines, T47D and MCF7. In addition, no expression of PD-L1 was detected in the non-tumorigenic breast epithelial cell line MCF 10A (Figure 1A). To explore the role of PD-L1 on major tumor progression hallmarks in TNBC cells, MDA-MB-231 cells were stably transduced with three different designs of all-in-one lentiviral particles containing in each vector a single gRNA targeting PD-L1 and Cas9 or CRISPR negative control non-targeting particles. GFP-positive cells were expanded in 96-well plates to generate pure clones (10 to 12 for each design). These clones were analyzed by western blotting to confirm PD-L1 knockout. As expected, the control CRISPR has no impact on PD-L1 expression in MDA-MB-231 cells (Figure 1B). Three clones from each design showed a complete knockout of PD-L1 (Figure 1C–E). The three pure clones were pooled together for each PD-L1 sgRNA design or negative control to obtain a better representative pool of pure clones. The generated pools of clones were named PD-L1.1 (pool of clones 2, 3, 8), PD-L1.2 (pool of clones 2, 5, 6), PD-L1.3 (pool of clones 2, 19, 21), and the negative control is named PD-L1 control (pool of clones B, C, F) (Figure 1F).

### 2.2. PD-L1 Knockout Decreases MDA-MB-231 Cells Proliferation, Colony Formation In Vitro, and Tumor Growth In Vivo

**Figure 2:**
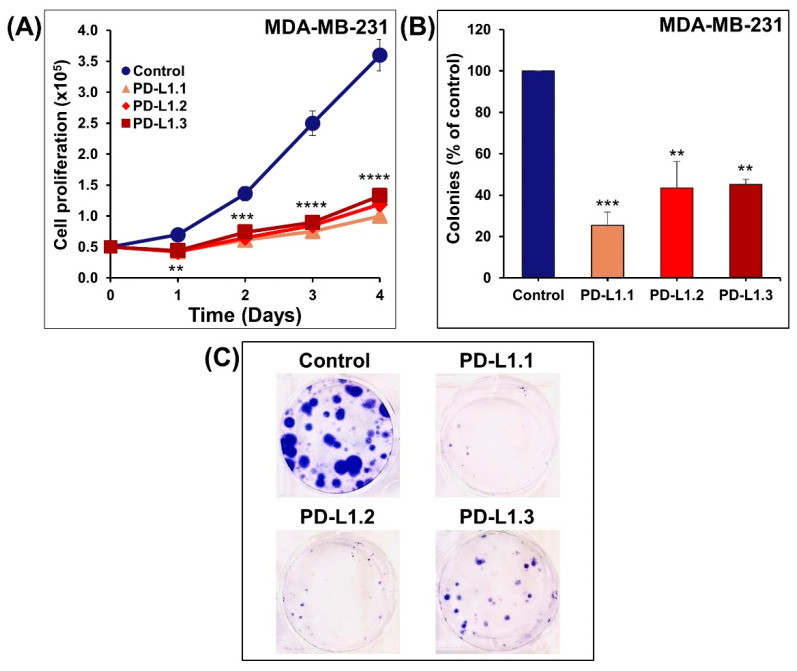
Impact of PD-L1 knockout on proliferation rate and colony growth of MDA-MB-231. (**A**) Control- and PD-L1 knockout cells were seeded into six-well plates for 1, 2, 3, and 4 days. The cells proliferation was determined using a cell counter as described in the Materials and Methods. (**B**,**C**) The control and knockout cells were seeded and kept to form colonies for 3 weeks after which the colonies were fixed, stained, and counted, as described in the Materials and Methods. Experiments were repeated three times. Shapes/Columns represent mean; bars represent S.E.M. ** *p* < 0.01, *** *p* < 0.001, **** *p* < 0.0001.

A high proliferation rate is a prominent hallmark of breast cancer and is significantly associated with the level of PD-L1 expression in breast cancer cells [18]. Therefore, MDA-MB-231 cell proliferation rates were determined for up to 4 days using a cell counter (Scepter™, MerckMillipore, Darmstadt, Germany). Figure 2A showed significantly decreased proliferation rates in PD-L1 knockout cells PD-L1.1, PD-L1.2, and PD-L1.3 compared to the control cells after incubation for 1, 2, 3, and 4 days. Consequently, we investigated the MDA-MB-231 PD-L1 knockout cells’ ability to form colonies. PD-L1.1, PD-L1.2, and PD-L1.3 cells were seeded into six-well plates at a density of 150 cells per well for three weeks to form colonies. The results showed a significantly decreased number of colonies in PD-L1.1, PD-L1.2, and PD-L1.3 compared to the control (Figure 2B,C). These findings imply that PD-L1 absence substantially limits breast cancer growth, which comes into agreement with Chen et al. who reported a decrease in TNBC MDA-MB-231 cell growth and colony formation in soft agar upon PD-L1 knockout using CRISPR/Cas9 or knockdown using siRNA [19]. Also, other studies showed that stable PD-L1 shRNAs knockdown significantly inhibited the cell proliferation of different cancer cells, including human gastric cancer cells SGC-7901 and AGS [20], murine ovarian cancer cells ID8agg, and melanoma cells B16 [21], in addition to human esophageal cancer cells Eca-109 [22]. Transient knockdown of PD-L1 by siRNA decreases the viability and colony formation of oral squamous cell carcinoma SAS and YD38 cells [23] and TNBC MDA-MB-231 [24]. Yu et al. (2020) obtained similar results and reported the inhibition in the cell viability and colony formation of H460 and H358 cells transduced with PD-L1-specific siRNA [25].

**Figure 3:**
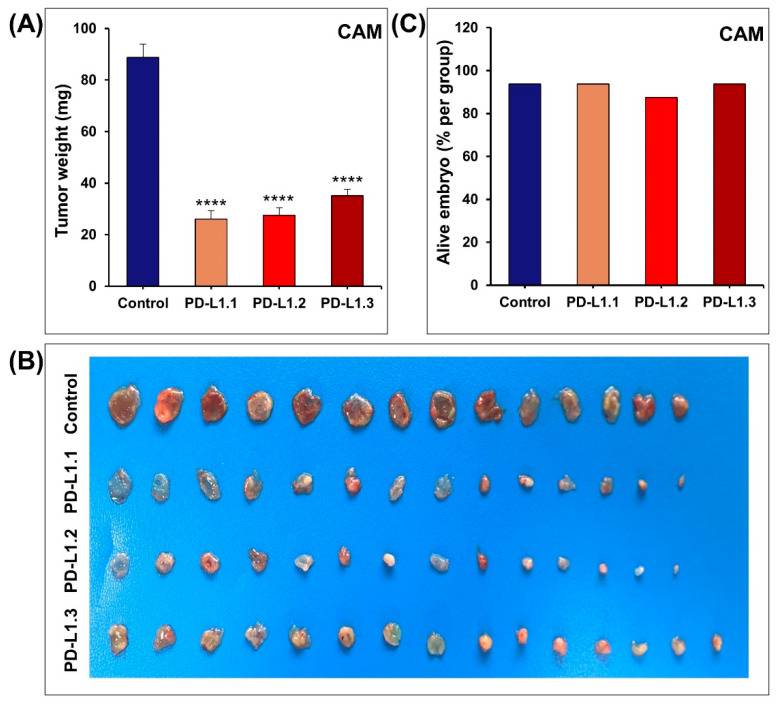
Impact of PD-L1 knockout on tumor growth in chick embryo CAM. (**A**) Tumor weight of the control and PD-L1 knockout cells. The cells were inoculated and allowed to grow on chick embryo CAM for 8 days. (**B**) Pictures of the tumors after extraction on Day 17. (**C**) Percentage of alive embryos in each group. Columns are means; bars are S.E.M. **** *p* < 0.0001.

To confirm the relevance of our in vitro data, the anticancer impact of PD-L1 knockout was investigated in vivo using the chick embryo CAM tumor growth assay. Cells grafted on the CAM at E09 were left to form tumors. At E17, tumors were retrieved from the upper CAM and weighed. In line with our in vitro findings, we found that PD-L1.1, PD-L1.2, and PD-L1.3 tumor xenografts were significantly smaller than control tumors by 70%, 69%, and 61%, respectively (Figure 3A,B). The impact of MDA-MB-231 tumors xenografts on chick embryos survival was evaluated by assessing the number of alive embryos in control and PD-L1 Knockout groups. There was no difference in the number of surviving embryos between all the groups at the end of the experiment (Figure 3C). These findings are in agreement with a recent article documenting the inhibitory effects of PD-L1 inhibitors (atezolizumab and avelumab) on MDA-MB-231 and A375 tumor growth in chick embryo CAM models without inducing significant toxicity on the chick embryos [26]. The inhibition of tumor growth in vivo by stable knockdown of PD-L1 has been reported in melanoma B16 tumors xenografted in NSG mice [21], gastric SGC-7901 tumors planted in nude mice [20], glioma xenografts in nude mice [27], and NSCLC H460 in BALB/c-nude mice [25].

### 2.3. PD-L1 Knockout Decreases MDA-MB-231 Cell Migration and Invasion In Vitro

**Figure 4:**
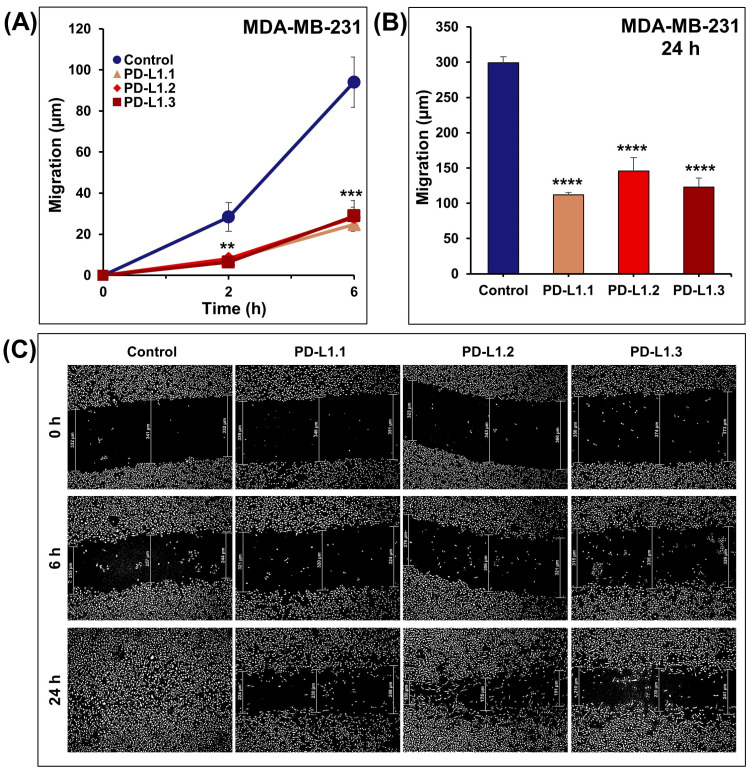
PD-L1 knockout significantly decreases MDA-MB-231 cell migration in vitro. Using wound healing assay, scratches were introduced to the adherent monolayer of control and PD-L1 knockout cells. (**A**,**B**) Migration distance was determined after 2, 6, and 24 h. (**C**) Representative pictures of the healing progress of the induced wounds at the indicated time points. Experiments were repeated at least three times. Columns or shapes are means; bars are S.E.M. ** *p* < 0.01. *** *p* < 0.001. **** *p* < 0.0001.

Cancer metastasis is a life-threatening event in which cell migration and invasion are critical steps. Here, we investigated the impact of PD-L1 knockout on MDA-MB-231 cell migration using the scratch wound-healing migration assay. As shown in Figure 4A–C, the migration of PD-L1 knockout MDA-MB-231 cells at 2, 6, and 24 h decreased significantly compared to the control cells. At 24 h, the wound was completely closed in MDA-MB-231 control. However, the three PD-L1 knockout cells failed to heal their scratch wounds.

**Figure 5:**
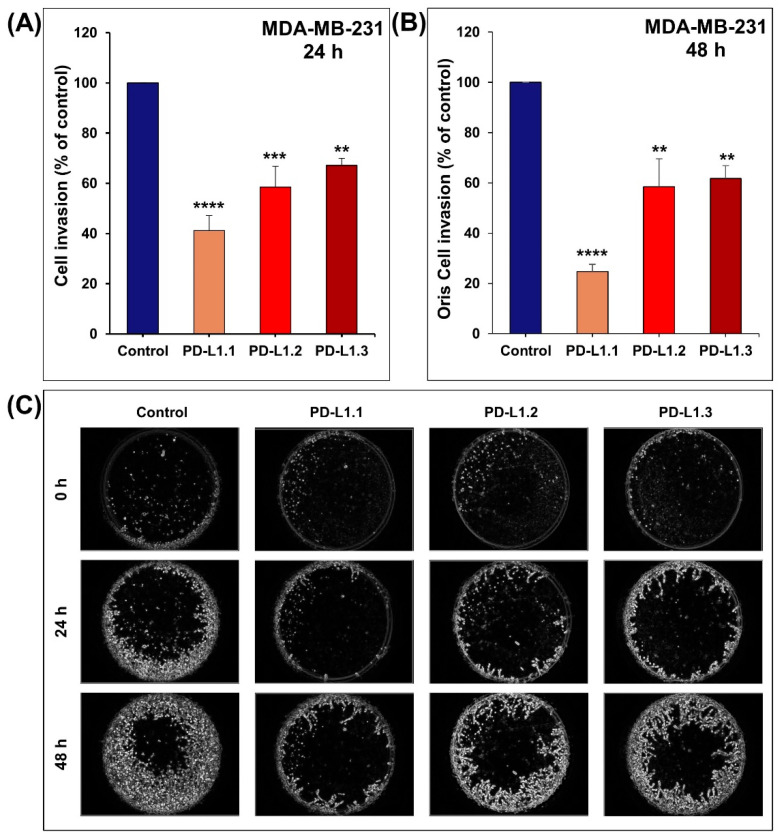
PD-L1 knockout significantly decreases MDA-MB-231 invasion in vitro. (**A**) Using Boyden Matrigel invasion chamber assay, control and PD-L1 knockout cells were seeded in the upper chamber for 24 h. The invading cells into the lower chamber were determined using CellTiter-Glo Luminescent. (**B**,**C**) Using Oris Matrigel invasion assay, invasion of the control and PD-L1 knockout cells into the detection zone was monitored for 24 and 48 h. Experiments were repeated at least 3 times. Columns are means; bars are S.E.M. ** *p* < 0.01. *** *p* < 0.001. **** *p* < 0.0001.

Next, we investigated the impact of PD-L1 knockout on the invasiveness of MDA-MB-231 cells compared to the control cells using the Boyden Chamber Matrigel invasion assay and the Oris™ Matrigel Cell Invasion Assay. We demonstrated in the two invasion assays that the invasiveness of the PD-L1 knockout cells decreased significantly compared to the control cells (Figure 5A–C). These data show that the PD-L1 protein is a strong promoter of breast cancer cell migration and invasion. This comes in agreement with the impact of PD-L1 knockdown on migration and invasion of gastric cancer SGC-7901 and AGS cells [20], breast cancer MDA-MB-231 cells [19,24], and oral squamous cell carcinoma SAS and YD38 cells [23]. Additionally, it has been reported in head and neck squamous carcinoma cell lines that PD-L1 overexpression significantly increases migration and invasion, while PD-L1 knockdown reduces the migration and invasion ability [28]. Similar findings were reported on migration and invasion of NSCLC cells upon overexpression of PD-L1 in H1299 and A549 cells and knockdown in H460 and H358 cells [25], migration of human esophageal cancer Eca-109 cells [22], and migration of glioblastoma multiforme [27]. On the contrary, PD-L1 knockout in osteosarcoma cells did not cause significant effects on cell migration and invasion [29]. Taken together, these data confirm the contribution of PD-L1 in tumor cell migration and invasion independently from PD-1 receptor interaction.

### 2.4. The Role of Signaling Pathways in PD-L1 Mediated Proliferation, Migration, Invasion, and Tumor Growth

To explore the various molecular pathways through which PD-L1 might contribute to the tumor progression of breast cancer cells, ERK, and Akt pathways, in addition to Rho GTPases, have been investigated in our PD-L1 knockout MDA-MB-231 cells.

#### 2.4.1. Impact of PD-L1 on ERK Signaling Pathway

**Figure 6:**
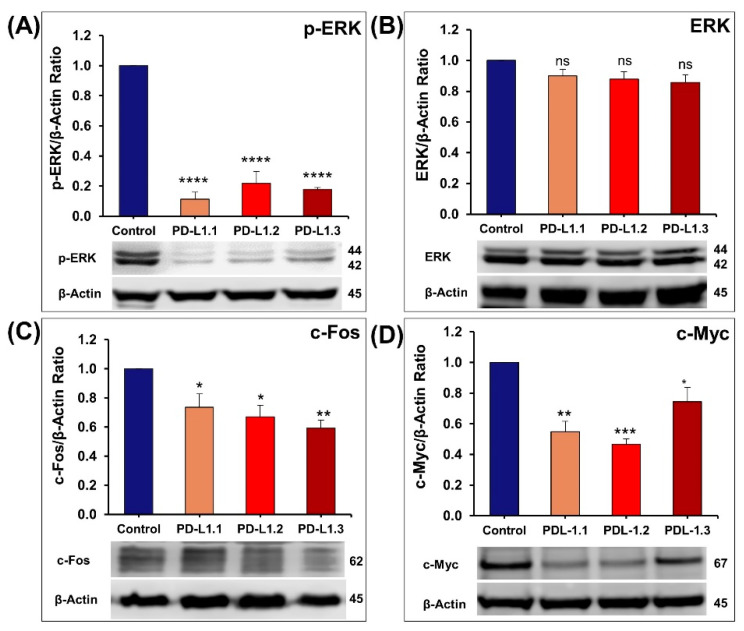
Quantification of the western blot showing the impact of PD-L1 knockout on the expression of p-ERK and total ERK (**A**,**B**), c-Fos (**C**), and c-Myc (**D**). Columns represent mean of at least three independent experiments; bars represent S.E.M. * *p* < 0.05. ** *p* < 0.01. *** *p* < 0.001. **** *p* < 0.0001. ns—non-significant.

Mitogen-activated protein kinase (MAPK) signaling pathways are critical for cancer cell survival, proliferation, differentiation, invasion, metastasis, and tumor growth [30]. The current study demonstrated that PD-L1 knockout significantly decreases ERK phosphorylation without impacting total ERK expression (Figure 6A,B). This comes in agreement with Geum et al., (2022), who reported that PD-L1 siRNA decreased p-ERK level in oral squamous cell carcinoma cells [23]. Increased phosphorylation of ERK was reported in glioblastoma multiforme upon PD-L1 overexpression [27]. Additionally, Passariello et al. (2019) reported that atezolizumab, an anti-PD-L1 mAb, showed inhibitory effects on p-ERK with a slight effect on p-P38 levels and no effect on the level of p-JNK in SK-BR-3 human breast tumor cells [31]. Chen et al reported that knockout or transient knockdown of PD-L1 in MDA-MB-231 had no impact on ERK phosphorylation [19]. However, treatment of MDA-MB-231 cells with atezolizumab revealed many affected genes related to the MAPK signaling pathway [32].

MAPKs, including ERK, p38, and JNK activation, increases the expression and the activity of AP-1 complex families like the Fos family (c-Fos, FosB, Fra-1, and Fra-2) [33,34]. c-Fos has an important role in tumor formation; it has been reported to participate in the regulation of proliferation, cell cycle progression, differentiation, and apoptosis [35,36]. In this context, we showed that the decrease in ERK phosphorylation in PD-L1 knockout cells was associated with a significant reduction in c-Fos expression (Figure 6C). Additionally, ERK1/2 phosphorylation activates many transcription factors such as CREB, c-Myc, and NFĸB, leading to the expression of genes encoding proteins that regulate key functions in cancer progression [30]. Myc is an oncoprotein that is highly expressed in TNBC and significantly associated with short overall survival [37]. Its activities are executed through transcriptional repression of cell cycle inhibitors p15, p21, and p27, which contributes to its effects on promoting proliferation and oncogenesis [38]. In this study, we demonstrated that PD-L1 knockout in MDA-MB-231 cells was also associated with a significant decrease in c-Myc expression (Figure 6D).

#### 2.4.2. Impact of PD-L1 on Akt Signaling Pathway

**Figure 7:**
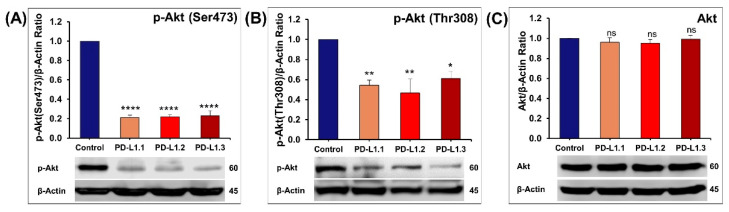
Quantification of the western blot showing the impact of PD-L1 knockout on the expression of p-Akt (Ser473) (**A**), p-Akt (Thr308) (**B**), and total Akt (**C**). Columns represent mean of at least three independent experiments; bars represent S.E.M. * *p* < 0.05. ** *p* < 0.01. **** *p* < 0.0001. ns—non-significant.

The Akt serine/threonine kinase, also known as protein kinase B (PKB), is the central node of the PI3K/Akt pathway that is a key regulator of cellular processes involved in cell metabolism, proliferation, survival, migration, and invasion. PDK1 induced the phosphorylation of Akt at Thr308. To achieve its full activation, Akt needs to be phosphorylated at Ser473 mainly by mTORC2 [39]. Phosphorylation of Akt at Ser473 has been reported to promote breast cancer metastasis [40,41]. The western-blot analysis shows that PD-L1 depletion in TNBC cells inhibits Akt phosphorylation at Ser473 and Thr308 (Figure 7A,B) without affecting the total Akt level (Figure 7C). In this context, it has been reported that PD-L1 overexpression increased the level of p-Akt in H1299 lung cancer cells [25] and in HT-29 and HCT-116 colorectal cancer cells [42]. In addition, the transient knockdown of PD-L1 in oral squamous cell carcinoma decreased the phosphorylation of Akt [23]. Similarly, decreased p-Akt was documented in pancreatic cancer and liver metastasis tissues in mice treated with anti-PD-L1 antibody [43]. The activation of the PI3K/Akt pathway by PD-L1 has also been reported in nasopharyngeal cancer [44] and breast cancer stem cells [45]. On the contrary, it has been reported that PD-L1 knockout or transient knockdown in MDA-MB-231 slightly increases the AKT phosphorylation [19].

The contribution of Akt and Erk signaling to tumor progression by inhibiting apoptosis and promoting cell proliferation, migration, and invasion is widely accepted. The complexity of the intrinsic crosstalk of MAPK with other signaling pathways, including the Akt pathway, is very challenging. It has been reported that the loss of phosphatase PTEN, the main inhibitor of Akt-phosphorylation, also leads to Ras/MAPK activation. The recent trend in clinical trials is to combine MAPK and Akt inhibitors [30]. Here, we demonstrated that PD-L1 is a key activator of the two major signaling pathways, MAPK and Akt. Encouraged by these results, we decided to look further into some of the downstream proteins, such as COX-2, survivin, and p21.

#### 2.4.3. Investigation of ERK and Akt Downstream Proteins

**Figure 8:**
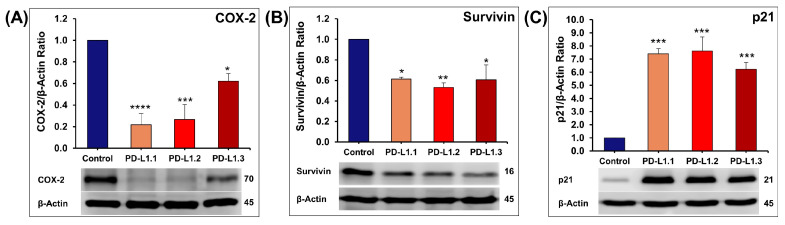
Quantification of the western blot showing the impact of PD-L1 knockout on the expression of COX-2 (**A**), Survivin (**B**), and p21 (**C**). Columns represent mean of at least three independent experiments; bars represent S.E.M. * *p* < 0.05. ** *p* < 0.01. *** *p* < 0.001. **** *p* < 0.0001.

COX-2, an established pro-inflammatory protein [46], was also described to modulate proliferation, apoptosis, and invasion, mainly in solid tumors [47]. It has been reported that COX-2 expression is correlated with increased phosphorylation of Akt [48] and phosphorylation of ERK [49]. Guo et al. (2001) reported that transcription factor c-Fos upregulates the expression of COX-2 [50]. In this context, we showed that the decrease in p-Akt, p-ERK, and its downstream factor c-Fos is accompanied by a reduction of COX-2 expression in the PD-L1 knockout cells (Figure 8A).

Survivin is the smallest member of the IAP family that possesses a potent anti-apoptotic activity. Its overexpression has been reported to be associated with cancer cell migration, invasion, and apoptosis resistance [51]. Overexpression of survivin is associated with poor prognosis. Survivin can be upregulated by Akt [52]. Hence, in this study, the decrease in Akt phosphorylation led to a statistically significant reduction in the expression of survivin (Figure 8B). These data are in agreement with the results showing evidence of survivin up-regulation by Akt and HIF–1α [53] and data reporting that PI3K regulates survivin expression through Akt activation [52]. The decrease in survivin seen in this study may also be linked to COX-2 level because it has been positively correlated with COX-2 level in breast cancer tissues [54] and NSCLC [55]. It has been reported that COX-2 antisense-derived NSCLC tumors have decreased survivin levels in contrast to the COX-2 sense-derived tumors that show elevated survivin [55]. Similar observations were reported on survivin in cells treated with COX-2 inhibitors [56,57].

p21 mediates cell cycle arrest in G1 phase by inhibiting the activity of cyclin/CDK2 complexes leading to the inhibition of pRb phosphorylation, and consequently, E2F transcription factors remain sequestered and unable to promote entry into S phase [58]. c-Myc is the most important suppressor of p21 transcription [59] in addition to Akt, which directly inhibits the cell cycle inhibitor p21Cip1/WAF1 [60,61,62]. In agreement, we demonstrate that inhibition of c-Myc expression and Akt-phosphorylation induced by PD-L1 knockout was associated with more than six folds increase in the expression of cyclin-dependent kinase (CDK) inhibitor p21Cip1/WAF1 (Figure 8C). Our results shed light on the implication of p21, COX-2, survivin, c-Myc, and c-Fos through ERK and Akt signaling pathways in the PD-L1-mediated cancer progression.

#### 2.4.4. Impact of PD-L1 on Rho GTPases Signaling Pathway

**Figure 9:**
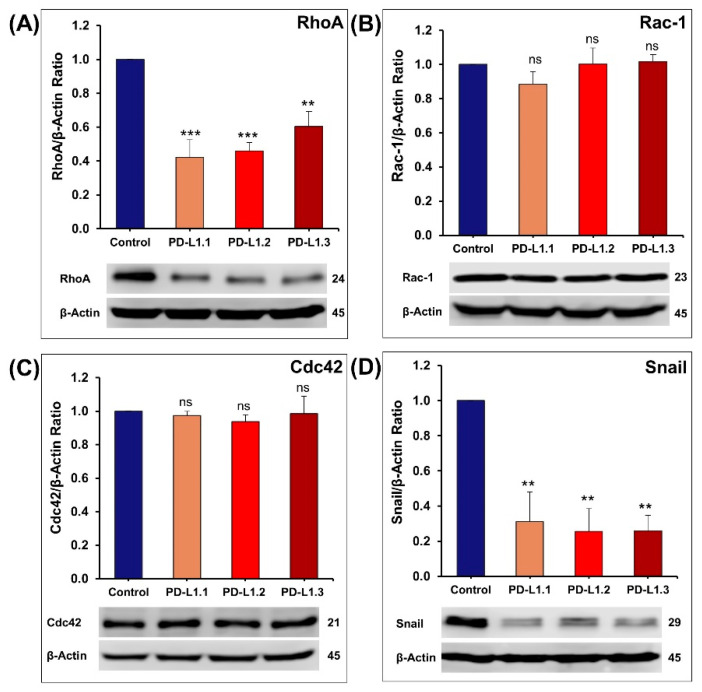
Quantification of western blot showing the effect of PD-L1 knockout on the expression of RhoA (**A**), Rac-1 (**B**), Cdc42 (**C**), and Snail (**D**). Experiments were repeated at least three times. Results are expressed as means ± S.E.M. ** *p* < 0.01. *** *p* < 0.001. ns—non-significant.

Rho GTPases overexpression has been reported in human tumors, including breast cancer [63,64]. Rho GTPases (RhoA, Rac, and Cdc42) play a major role in breast cancer cell proliferation, adhesion, migration, invasion, and metastasis [65]. RhoA stimulates actin-myosin contractility, resulting in the formation of stress fibers and focal adhesions and providing cell–extracellular matrix (ECM) anchoring points needed to pull the cell body in the direction of movement. Rac1 controls the formation of lamellipodia at the leading edge of the migrating cell. Finally, Cdc42 signaling leads to the formation of filopodia and maintains the cellular polarization required for directional migration [65]. Therefore, we have decided to investigate whether PD-L1 regulation of MDA-MB-231 cancer cell proliferation, migration, and invasion involves the Rho GTPase signaling cascade. We demonstrated that PD-L1 knockout was associated with a significant decrease in RhoA protein expression without any impact on Rac1 and cdc42 proteins expression (Figure 9A–C). Eichberger et al. (2020) reported a PD-L1-dependent gene regulation of Rho-GTPases Rho and Rac1 in head and neck cancer cells [28]. It has also been reported that Rho inhibition by siRNA reduces MDA-MB-231 cancer cell proliferation and invasion [66]. Liberto et al. (2002) reported that RhoA is required for G1 to S progression in mammary epithelial cells by repression of p21 (Waf1/Cip1) [67]. In this context, we suggest that the increased expression of p21 (Waf1/Cip1) observed in our MDA-MB-231 PD-L1 knockout cells is at least in part due to the decrease in the expression of RhoA.

Snail confers a migratory and invasive ability essential for malignant cells MDA-MB-231 to disseminate and form metastasis [68]. It was reported that Snail contributes to the progression of breast tumors through the regulation of RhoA [69]. Here, we showed that the decrease in RhoA expression in PD-L1 knockout MDA-MB-231 cells is also associated with a decrease in the expression of Snail (Figure 9D). This is in agreement with a recent article reporting that PD-L1 knockout or knockdown in MDA-MB-231 caused a reduction in snail level without affecting its mRNA level [19]. The study by Kim et al. (2016) also supports our findings by reporting that in pulmonary adenocarcinoma, the EMT marker snail expression level correlates positively with PD-L1 expression [70].

**Figure 10:**
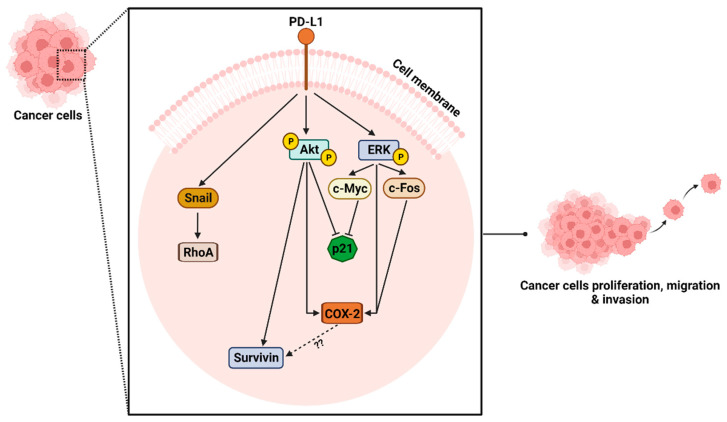
Diagram highlighting the potential downstream signaling of PD-L1 in TNBC. Created with BioRender.com.

In summary, we have demonstrated that PD-L1 knockout in the human TNBC MDA-MB-231 cells significantly decreased cell proliferation, colony formation, migration and invasion in vitro and tumor growth in chick embryo CAM assay in vivo. As shown in Figure 10, our results revealed that these effects were partially mediated via downregulating the ERK and Akt activity and the expression of Snail and RhoA in addition to some of their downstream proteins. This study provides new insights into understanding the PD-1 independent role of PD-L1 in breast cancer.

## 3. Materials and Methods

### 3.1. Cell Culture and Antibodies

Human triple-negative breast cancer (TNBC) cells MDA-MB-231 were maintained in DMEM (Hyclone, Cramlington, UK) supplemented with antibiotics (penicillin 50 U/mL; streptomycin 50 µg/mL) and 10% fetal bovine serum (Hyclone, Cramlington, UK) at 37 °C. The culture medium was changed every three days, and cells were passaged once a week when the culture reached 95% confluence. Cell viability measured using trypan blue dye exclusion was higher than 99% in all experiments. All in vitro experiments were repeated at least three times. Antibodies to PD-L1, Akt, phospho-Akt, phospho-ERK, Snail, p21, and β-tubulin were obtained from Cell Signaling Technology (Cell Signaling, Beverly, MA, USA). Antibodies to ERK, Survivin, RhoA, Rac1, Cdc42, c-Fos, c-Myc, and β-actin HRP were obtained from Santa Cruz Biotechnology, Inc. (Santa Cruz, CA, USA). COX2 antibody was obtained from Abcam (Cambridge, UK).

### 3.2. Establishment of Stable PD-L1 Knockout Clones in Breast Cancer Cells

MDA-MB-231 cells were seeded at a density of 4000 cells/well into 96-well plates in the presence of the serum and allowed to attach for 24 h. Cells were transduced in serum-free media for 24 h with three different designs of all-in-one lentiviral particles containing a single gRNA targeting PD-L1 and Cas9 in each vector or CRISPR negative control particles (Transomic technologies, Huntsville, AB, USA). Then, cells were incubated for another 72 h in complete media. Each transduction was transferred into three p96 plates at a density of 40 cells per plate to have one cell/well. Multiple pure GFP positive clones were expanded, harvested, and prepared for western blot analyses of PD-L1 knockout.

### 3.3. Impact of PD-L1 Knockout on Cell Proliferation

Control- and PD-L1 knockout pool of clones (PD-L1.1, PD-L1.2, and PD-L1.3) were plated at the density of 50,000 cells into six-well tissue culture dishes supplemented with 10% FBS. At the indicated time, cells were trypsinized, collected in 1 mL of medium, and counted at an appropriate dilution every day for four consecutive days using a cell counter (Scepter™, MerckMillipore, Darmstadt, Germany).

### 3.4. Colony Formation Assay

Control- and PD-L1-transduced cells were seeded into six-well plates at a density of 150 cells per well for three weeks. Following that, colonies were washed three times with PBS, fixed, and stained for 2 h with 0.25% crystal violet dissolved in (*v/v*) distilled water/methanol. Colonies were again washed three times with PBS then photographed. The percentages of colonies with more than 50 cells were determined in PD-L1-transduced colonies compared to the control-transduced colonies assumed to be 100%.

### 3.5. Impact on Cellular Migration Using Wound Healing Assay

The control, compared to PD-L1- knockout cells (0.75 × 10^6^ cells), was grown in twelve-well tissue culture dishes until confluence. A scrape was made through the confluent monolayer using a 200 µL tip. Afterwards, the plates were washed twice and incubated at 37 °C in fresh media containing 10% FBS. At the top side of each dish, two random places were marked where the width of the wound was measured using an inverted microscope at objective 4x (Olympus, Tokyo, Japan). Migration was expressed as the mean ± SEM of the difference between the measurements at time zero and the 2, 6, and 24 h time periods considered.

### 3.6. Boyden Chamber Matrigel Invasion Assay

The invasiveness of the control, compared to PD-L1-knockout cells, was tested using Corning BioCoat™ Matrigel Invasion Chamber (8-µm pore size) (Corning, Bedford, MA, USA) according to manufacturer’s protocol. Briefly, cells (1 × 10^5^) in 0.5 mL of media serum-free were seeded into the upper chambers of the system. The bottom chamber in the system was filled with media supplemented with 10% FBS as a chemo-attractant and then incubated at 37 °C for 24 h. Non-invasive cells were removed from the upper surface of the filter by gently rubbing the area with a cotton swab. Cells that invaded the Matrigel and passed through the 8 µm pores of the insert were detected using CellTiter-Glo^®^ Luminescent Cell Viability assay (Promega Corporation, Madison, WI, USA) as previously described [71]. The impact of PD-L1 knockout on cellular invasion was presented as a percentage (%) by comparing the invasiveness of PD-L1- knockout cells to the control cells.

### 3.7. The Oris™ Matrigel Cell Invasion Assay

The invasiveness of PD-L1 knockout cells compared to control cells was also investigated using a 3-dimensional extracellular Matrigel matrix (Corning, Bedford, UK). Cells were seeded at 100,000 cells per well and allowed to attach overnight onto a 96-well plate coated with Matrigel at the concentration of 3.5 mg/mL. Once the cells formed a confluent monolayer, the silicone stoppers were removed. Wells were washed twice with PBS and then covered with 40 µL of Matrigel at the concentration of 5.5 to 6 mg/mL and incubated at 37 °C in the incubator for 45 min and then incubated in complete media for 24 and 48 h. The invasiveness of the MDA-MB-231 cells was assessed using an Olympus fluorescence microscope (Olympus, Tokyo, Japan). Representative figures were taken at 0, 24, and 48 h and converted to black and white. Using Image J, the density of the invasive cells at 48 h was measured and compared to the control cells density, the invasiveness of which is assumed to be 100%.

### 3.8. Chick Embryo CAM Tumor Growth Assay

Fertilized White Leghorn eggs were incubated at 37.5 °C and 50% humidity. On the embryonic day 3 (E3), the chorioallantoic membrane (CAM) was dropped by drilling a small hole through the eggshell into the air sac, and a 1-cm^2^ window was cut in the eggshell above the CAM. At day 9 (E9), control- and PD-L1-knockout MDA-MB-231 cells were trypsinized, washed with complete medium, suspended in an 80% Matrigel^®^ Matrix (Corning, Bedford, UK) at the density of 1 × 10^6^ cells per 100 μL, and added onto the CAM of each egg, for a total of 15 to 16 eggs per cell line. At embryonic day 17 (E17), embryos were humanely euthanized by a topical addition of 10–30 μL pentobarbitone sodium (300 mg/mL, Jurox, Auckland, New Zealand). The upper portions of the CAM were removed, transferred in PBS, and then the tumors were carefully cut away from normal CAM tissues and weighted. Alive embryos were determined by checking the voluntary movements of the embryos and the integrity and pulsation of the blood vessels.

### 3.9. Western-Blot Analysis

Total cellular proteins from PD-L1 knockout and control cells were isolated using RIPA buffer (25 mM Tris.HCl pH 7.6), 1% nonidet P-40, 1% sodium deoxycholate, 0.1% SDS, 0.5% protease inhibitors cocktail (Sigma, Steinheim, Germany), 1% PMSF, and 1% phosphatase inhibitors cocktail (Thermo Scientific, Rockford, IL, USA). The whole-cell lysate was recovered by centrifugation at 14,000 rpm for 20 min at 4 °C to remove insoluble material and 20 µg of proteins were separated by SDS gel for identification of PD-L1 targets expression and activities. After electrophoresis, the proteins were transferred on a nitrocellulose membrane, blocked with 5% non-fat milk in TBST (TBS and 0.05% Tween 20), and probed with primary, β-tubulin, and β-actin antibodies overnight at 4 °C. The blots were washed and exposed to secondary antibodies. Immunoreactive bands of PD-L1, Akt, p-Akt(Ser473), p-Akt(Thr308), ERK, p-ERK, p21, COX-2, Survivin, c-Fos, c-Myc, Rho A, Snail, Rac1, Cdc42, β-tubulin, and β-actin were detected using ECL chemiluminescent substrate (Thermo Fisher Scientific, Waltham, MA, USA), and chemiluminescence was detected using the LiCOR C-DiGit blot scanner (LI-COR Biotechnology, Lincoln, NE, USA). Densitometry analysis was performed using an HP Deskjet F4180 Scanner with ImageJ software. The intensities of the bands were normalized to the intensities of the corresponding β-tubulin or β-actin bands.

### 3.10. Statistics

All in vitro experiments were repeated at least three times, and results are expressed as means ± SEM of the indicated data. Statistical analysis was performed with GraphPad Prism7 (La Jolla, CA, USA). The difference between experimental and control values was assessed by ANOVA followed by Dunnett’s post-hoc multiple comparisons test. * *p* <0.05. ** *p* < 0.01. *** *p* < 0.001. **** *p* < 0.0001 indicates a significant difference.

## Data Availability

Not applicable.

## References

[1] Sung H., Ferlay J., Siegel R.L., Laversanne M., Soerjomataram I., Jemal A., Bray F. (2021). Global Cancer Statistics 2020: GLOBOCAN Estimates of Incidence and Mortality Worldwide for 36 Cancers in 185 Countries. CA Cancer J. Clin..

[2] Yin L., Duan J.-J., Bian X.-W., Yu S.-C. (2020). Triple-negative breast cancer molecular subtyping and treatment progress. Breast Cancer Res..

[3] Ensenyat-Mendez M., Llinàs-Arias P., Orozco J.I.J., Íñiguez-Muñoz S., Salomon M.P., Sesé B., DiNome M.L., Marzese D.M. (2021). Current Triple-Negative Breast Cancer Subtypes: Dissecting the Most Aggressive Form of Breast Cancer. Front. Oncol..

[4] Cirqueira M.B., Mendonça C.R., Noll M., Soares L.R., de Paula Carneiro Cysneiros M.A., Paulinelli R.R., Moreira M.A.R., Freitas-Junior R. (2021). Prognostic Role of PD-L1 Expression in Invasive Breast Cancer: A Systematic Review and Meta-Analysis. Cancers.

[5] Wu Y., Chen W., Xu Z.P., Gu W. (2019). PD-L1 Distribution and Perspective for Cancer Immunotherapy—Blockade, Knockdown, or Inhibition. Front. Immunol..

[6] Lastwika K.J., Wilson W., Li Q.K., Norris J., Xu H., Ghazarian S.R., Kitagawa H., Kawabata S., Taube J.M., Yao S. (2016). Control of PD-L1 Expression by Oncogenic Activation of the AKT-mTOR Pathway in Non–Small Cell Lung Cancer. Cancer Res..

[7] Luo M., Xia Y., Wang F., Zhang H., Su D., Su C., Yang C., Wu S., An S., Lin S. (2021). PD0325901, an ERK inhibitor, enhances the efficacy of PD-1 inhibitor in non-small cell lung carcinoma. Acta Pharm. Sin. B.

[8] Rom-Jurek E.-M., Kirchhammer N., Ugocsai P., Ortmann O., Wege A.K., Brockhoff G. (2018). Regulation of Programmed Death Ligand 1 (PD-L1) Expression in Breast Cancer Cell Lines In Vitro and in Immunodeficient and Humanized Tumor Mice. Int. J. Mol. Sci..

[9] Zheng Y., Fang Y., Li J. (2019). PD-L1 expression levels on tumor cells affect their immunosuppressive activity. Oncol. Lett..

[10] Escors D., Gato-Cañas M., Zuazo M., Arasanz H., García-Granda M.J., Vera R., Kochan G. (2018). The Intracellular Signalosome of PD-L1 in Cancer Cells. Signal Transduct. Target. Ther..

[11] Zou W., Wolchok J.D., Chen L. (2016). PD-L1 (B7-H1) and PD-1 pathway blockade for cancer therapy: Mechanisms, response biomarkers, and combinations. Sci. Transl. Med..

[12] Makuku R., Khalili N., Razi S., Keshavarz-Fathi M., Rezaei N. (2021). Current and Future Perspectives of PD-1/PDL-1 Blockade in Cancer Immunotherapy. J. Immunol. Res..

[13] Han Y., Liu D., Li L. (2020). PD-1/PD-L1 Pathway: Current Researches in Cancer. Am. J. Cancer Res..

[14] Matikas A., Zerdes I., Lövrot J., Richard F., Sotiriou C., Bergh J., Valachis A., Foukakis T. (2019). Prognostic Implications of PD-L1 Expression in Breast Cancer: Systematic Review and Meta-analysis of Immunohistochemistry and Pooled Analysis of Transcriptomic Data. Clin. Cancer Res..

[15] Basu G.D., Ghazalpour A., Gatalica Z., Anderson K.S., McCullough A.E., Spetzer D.B., Pockaj B.A. (2014). Expression of novel immunotherapeutic targets in triple-negative breast cancer. J. Clin. Oncol..

[16] Li M., Li A., Zhou S., Xu Y., Xiao Y., Bi R., Yang W. (2018). Heterogeneity of PD-L1 expression in primary tumors and paired lymph node metastases of triple negative breast cancer. BMC Cancer.

[17] Liu L., Shen Y., Zhu X., Lv R., Li S., Zhang Z., Shi Y.G., Tan L. (2018). ERα is a negative regulator of PD-L1 gene transcription in breast cancer. Biochem. Biophys. Res. Commun..

[18] Ghebeh H., Tulbah A., Mohammed S., Elkum N., Bin Amer S.M., Al-Tweigeri T., Dermime S. (2007). Expression of B7-H1 in breast cancer patients is strongly associated with high proliferative Ki-67-expressing tumor cells. Int. J. Cancer.

[19] Chen C., Li S., Xue J., Qi M., Liu X., Huang Y., Hu J., Dong H., Ling K. (2021). PD-L1 Tumor-Intrinsic Signaling and Its Therapeutic Implication in Triple-Negative Breast Cancer. JCI Insight..

[20] Li J., Chen L., Xiong Y., Zheng X., Xie Q., Zhou Q., Shi L., Wu C., Jiang J., Wang H. (2017). Knockdown of PD-L1 in Human Gastric Cancer Cells Inhibits Tumor Progression and Improves the Cytotoxic Sensitivity to CIK Therapy. Cell. Physiol. Biochem..

[21] Clark C.A., Gupta H.B., Sareddy G., Pandeswara S., Lao S., Yuan B., Drerup J.M., Padron A., Conejo-Garcia J., Murthy K. (2016). Tumor-Intrinsic PD-L1 Signals Regulate Cell Growth, Pathogenesis, and Autophagy in Ovarian Cancer and Melanoma. Cancer Res..

[22] Chen L., Xiong Y., Li J., Zheng X., Zhou Q., Turner A., Wu C., Lu B., Jiang J. (2017). PD-L1 Expression Promotes Epithelial to Mesenchymal Transition in Human Esophageal Cancer. Cell. Physiol. Biochem..

[23] Geum D.-H., Hwang D.-S., Lee C.-H., Cho S.-D., Jang M.-A., Ryu M.-H., Kim U.-K. (2022). PD-L1 Expression Correlated with Clinicopathological Factors and Akt/Stat3 Pathway in Oral SCC. Life.

[24] Lotfinejad P., Kazemi T., Safaei S., Amini M., Roshani Asl E., Baghbani E., Sandoghchian Shotorbani S., Jadidi Niaragh F., Derakhshani A., Abdoli Shadbad M. (2021). PD-L1 silencing inhibits triple-negative breast cancer development and upregulates T-cell-induced pro-inflammatory cytokines. Biomed. Pharmacother..

[25] Yu W., Hua Y., Qiu H., Hao J., Zou K., Li Z., Hu S., Guo P., Chen M., Sui S. (2020). PD-L1 promotes tumor growth and progression by activating WIP and β-catenin signaling pathways and predicts poor prognosis in lung cancer. Cell Death Dis..

[26] Wang Y., Rousset X., Prunier C., Garcia P., Dosda E., Leplus E., Viallet J. (2022). PD-1/PD-L1 Checkpoint Inhibitors Are Active in the Chicken Embryo Model and Show Antitumor Efficacy In Ovo. Cancers.

[27] Qiu X.Y., Hu D.X., Chen W.-Q., Chen R.Q., Qian S.R., Li C.Y., Li Y.J., Xiong X.X., Liu D., Pan F. (2018). PD-L1 confers glioblastoma multiforme malignancy via Ras binding and Ras/Erk/EMT activation. Biochim. Et Biophys. Acta (BBA)-Mol. Basis Dis..

[28] Eichberger J., Schulz D., Pscheidl K., Fiedler M., Reichert T., Bauer R., Ettl T. (2020). PD-L1 Influences Cell Spreading, Migration and Invasion in Head and Neck Cancer Cells. Int. J. Mol. Sci..

[29] Liao Y., Chen L., Feng Y., Shen J., Gao Y., Cote G., Choy E., Harmon D., Mankin H., Hornicek F. (2017). Targeting programmed cell death ligand 1 by CRISPR/Cas9 in osteosarcoma cells. Oncotarget.

[30] Braicu C., Buse M., Busuioc C., Drula R., Gulei D., Raduly L., Rusu A., Irimie A., Atanasov A.G., Slaby O. (2019). A Comprehensive Review on MAPK: A Promising Therapeutic Target in Cancer. Cancers.

[31] Passariello M., D’Alise A.M., Esposito A., Vetrei C., Froechlich G., Scarselli E., Nicosia A., De Lorenzo C. (2019). Novel Human Anti-PD-L1 mAbs Inhibit Immune-Independent Tumor Cell Growth and PD-L1 Associated Intracellular Signalling. Sci. Rep..

[32] Saleh R., Taha R.Z., Sasidharan Nair V., Alajez N.M., Elkord E. (2019). PD-L1 Blockade by Atezolizumab Downregulates Signaling Pathways Associated with Tumor Growth, Metastasis, and Hypoxia in Human Triple Negative Breast Cancer. Cancers.

[33] Gazon H., Barbeau B., Mesnard J.-M., Peloponese J.-M. (2018). Hijacking of the AP-1 Signaling Pathway during Development of ATL. Front. Microbiol..

[34] Monje P., Hernández-Losa J., Lyons R.J., Castellone M.D., Gutkind J.S. (2005). Regulation of the Transcriptional Activity of c-Fos by ERK. J. Biol. Chem..

[35] You L., Ren X., Du Y., Zhao W., Cui M., Chen G., Zhao Y. (2016). c-Fos/ERK promotes the progression from pancreatic intraepithelial neoplasia to pancreatic ductal adenocarcinoma. Oncol. Rep..

[36] Angel P., Karin M. (1991). The role of Jun, Fos and the AP-1 complex in cell-proliferation and transformation. Biochim. Et Biophys. Acta (BBA)-Rev. Cancer.

[37] Gupta N., Jung K., Wu C., Alshareef A., Alqahtani H., Damaraju S., Mackey J.R., Ghosh S., Sabri S., Abdulkarim B.S. (2017). High Myc expression and transcription activity underlies intra-tumoral heterogeneity in triple-negative breast cancer. Oncotarget.

[38] Gartel A.L., Shchors K. (2003). Mechanisms of c-myc-mediated transcriptional repression of growth arrest genes. Exp. Cell Res..

[39] Hinz N., Jücker M. (2019). Distinct functions of AKT isoforms in breast cancer: A comprehensive review. Cell Commun. Signal..

[40] Qiao M., Iglehart J.D., Pardee A.B. (2007). Metastatic Potential of 21T Human Breast Cancer Cells Depends on Akt/Protein Kinase B Activation. Cancer Res.

[41] Song M., Bode A.M., Dong Z., Lee M.-H. (2019). AKT as a Therapeutic Target for Cancer. Cancer Res..

[42] Wei F., Zhang T., Deng S.-C., Wei J.-C., Yang P., Wang Q., Chen Z.-P., Li W.-L., Chen H.-C., Hu H. (2019). PD-L1 promotes colorectal cancer stem cell expansion by activating HMGA1-dependent signaling pathways. Cancer Lett..

[43] Zhao L., Li C., Liu F., Zhao Y., Liu J., Hua Y., Liu J., Huang J., Ge C. (2017). A blockade of PD-L1 produced antitumor and antimetastatic effects in an orthotopic mouse pancreatic cancer model via the PI3K/AkT/MTOR signaling pathway. OncoTargets Ther..

[44] Fei Z., Deng Z., Zhou L., Li K., Xia X., Xie R. (2019). PD-L1 Induces Epithelial-Mesenchymal Transition in Nasopharyngeal Carcinoma Cells Through Activation of the PI3K/AKT Pathway. Oncol. Res. Featur. Preclin. Clin. Cancer Ther..

[45] Almozyan S., Colak D., Mansour F., Alaiya A., Al-Harazi O., Qattan A., Al-Mohanna F., Al-Alwan M., Ghebeh H. (2017). PD-L1 promotes OCT4 and Nanog expression in breast cancer stem cells by sustaining PI3K/AKT pathway activation. Int. J. Cancer.

[46] Hashemi Goradel N., Najafi M., Salehi E., Farhood B., Mortezaee K. (2019). Cyclooxygenase-2 in Cancer: A Review. J. Cell Physiol..

[47] Sobolewski C., Cerella C., Dicato M., Ghibelli L., Diederich M. (2010). The Role of Cyclooxygenase-2 in Cell Proliferation and Cell Death in Human Malignancies. Int. J. Cell Biol..

[48] A Glynn S., Prueitt R.L., A Ridnour L., Boersma B.J., Dorsey T.M., A Wink D., E Goodman J., Yfantis H.G., Lee D.H., Ambs S. (2010). COX-2 activation is associated with Akt phosphorylation and poor survival in ER-negative, HER2-positive breast cancer. BMC Cancer.

[49] Chi F., Wu R., Jin X., Jiang M., Zhu X. (2016). HER2 induces cell proliferation and invasion of non-small-cell lung cancer by upregulating COX-2 expression via MEK/ERK signaling pathway. OncoTargets Ther..

[50] Guo Y.-S., Hellmich M.R., Wen X.D., Townsend C.M. (2001). Activator Protein-1 Transcription Factor Mediates Bombesin-stimulated Cyclooxygenase-2 Expression in Intestinal Epithelial Cells. J. Biol. Chem..

[51] McKenzie J.A., Liu T., Goodson A.G., Grossman D. (2010). Survivin Enhances Motility of Melanoma Cells by Supporting Akt Activation and α5 Integrin Upregulation. Cancer Res..

[52] Zhao P., Meng Q., Liu L.-Z., You Y.-P., Liu N., Jiang B.-H. (2010). Regulation of survivin by PI3K/Akt/p70S6K1 pathway. Biochem. Biophys. Res. Commun..

[53] Sun X.-P., Dong X., Lin L., Jiang X., Wei Z., Zhai B., Sun B., Zhang Q., Wang X., Jiang H. (2013). Up-regulation of survivin by AKT and hypoxia-inducible factor 1α contributes to cisplatin resistance in gastric cancer. FEBS J..

[54] Barnes N., Haywood P., Flint P., Knox W.F., Bundred N.J. (2006). Survivin expression in in situ and invasive breast cancer relates to COX-2 expression and DCIS recurrence. Br. J. Cancer.

[55] Krysan K., Merchant F.H., Zhu L., Dohadwala M., Luo J., Lin Y., Heuze-Vourc'H N., Põld M., Seligson D., Chia D. (2003). COX-2-dependent stabilization of survivin in non-small cell lung cancer. FASEB J..

[56] Sato A., Mizobuchi Y., Nakajima K., Shono K., Fujihara T., Kageji T., Kitazato K., Matsuzaki K., Mure H., Kuwayama K. (2017). Blocking COX-2 induces apoptosis and inhibits cell proliferation via the Akt/survivin- and Akt/ID3 pathway in low-grade-glioma. J. Neuro-Oncol..

[57] Li J., Hao Q., Cao W., Vadgama J.V., Wu Y. (2018). Celecoxib in breast cancer prevention and therapy. Cancer Manag. Res..

[58] Al Bitar S., Gali-Muhtasib H. (2019). The Role of the Cyclin Dependent Kinase Inhibitor P21cip1/Waf1 in Targeting Cancer: Molecular Mechanisms and Novel Therapeutics. Cancers.

[59] Madden S.K., de Araujo A.D., Gerhardt M., Fairlie D.P., Mason J.M. (2021). Taking the Myc out of cancer: Toward therapeutic strategies to directly inhibit c-Myc. Mol. Cancer.

[60] Suh E.-J., Kim Y.-J., Kim S.H. (2009). Protein phosphatase 2Cγ regulates the level of p21Cip1/WAF1 by Akt signaling. Biochem. Biophys. Res. Commun..

[61] Zhou B.P., Liao Y., Xia W., Spohn B., Lee M.-H., Hung M.-C. (2001). Cytoplasmic localization of p21Cip1/WAF1 by Akt-induced phosphorylation in HER-2/neu-overexpressing cells. Nat. Cell Biol..

[62] Xiao B.-D., Zhao Y.-J., Jia X.-Y., Wu J., Wang Y.-G., Huang F. (2020). Multifaceted p21 in carcinogenesis, stemness of tumor and tumor therapy. World J. Stem Cells.

[63] Orgaz J., Herraizy C., Sanz-Moreno V. (2014). Rho GTPases modulate malignant transformation of tumor cells. Small GTPases.

[64] Svensmark J.H., Brakebusch C. (2019). Rho GTPases in Cancer: Friend or Foe?. Oncogene.

[65] Humphries B., Wang Z., Yang C. (2020). Rho GTPases: Big Players in Breast Cancer Initiation, Metastasis and Therapeutic Responses. Cells.

[66] Pillé J.-Y., Denoyelle C., Varet J., Bertrand J.-R., Soria J., Opolon P., Lu H., Pritchard L.-L., Vannier J.-P., Malvy C. (2005). Anti-RhoA and Anti-RhoC siRNAs Inhibit the Proliferation and Invasiveness of MDA-MB-231 Breast Cancer Cells in Vitro and in Vivo. Mol. Ther..

[67] Liberto M., Cobrinik D., Minden A. (2002). Rho regulates p21CIP1, cyclin D1, and checkpoint control in mammary epithelial cells. Oncogene.

[68] Wang Y., Shi J., Chai K., Ying X., Zhou B. (2013). The Role of Snail in EMT and Tumorigenesis. Curr. Cancer Drug Targets.

[69] Zhang A., Wang Q., Han Z., Hu W., Xi L., Gao Q., Wang S., Zhou J., Xu G., Meng L. (2013). Reduced expression of Snail decreases breast cancer cell motility by downregulating the expression and inhibiting the activity of RhoA GTPase. Oncol. Lett..

[70] Kim S., Koh J., Kim M.-Y., Kwon D., Go H., A Kim Y., Jeon Y.K., Chung D.H. (2016). PD-L1 expression is associated with epithelial-to-mesenchymal transition in adenocarcinoma of the lung. Hum. Pathol..

[71] Al-Azawi A., Sulaiman S., Arafat K., Yasin J., Nemmar A., Attoub S. (2021). Impact of Sodium Dichloroacetate Alone and in Combination Therapies on Lung Tumor Growth and Metastasis. Int. J. Mol. Sci..

